# Serum Sickness-Like Reaction Associated With Acute Hepatitis B in a Previously Vaccinated Adult Male

**DOI:** 10.7759/cureus.14742

**Published:** 2021-04-28

**Authors:** Rahul Gupta, Ibukun Fakunle, Varun Samji, Elizabeth B Hale

**Affiliations:** 1 Internal Medicine-Pediatrics, Michigan State University/Hurley Medical Center, Flint, USA; 2 Internal Medicine-Pediatrics, Michigan State University, Flint, USA; 3 Internal Medicine, Michigan State University/Hurley Medical Center, Flint, USA; 4 Infectious Disease, Michigan State University/Hurley Medical Center, Flint, USA

**Keywords:** hepatitis, serum sickness, serum sickness-like reaction

## Abstract

Serum sickness is a well-known immune complex deposition phenomenon, occurring as a reaction to proteins in antiserum from a non-human animal source. Serum sickness-like reaction (SSLR), typically associated with drugs and vaccines, sometimes occurs with acute hepatitis B infection and poses a diagnostic dilemma for clinicians, as other viral syndromes, vasculitic processes, and autoimmune conditions can have similar presentations. We present a 36-year-old intravenous drug user, with confirmed records of hepatitis B immunization, who presented with multi-joint pain, joint swelling, and a skin rash. There is a paucity of cases in the literature reporting occurrence of serum sickness-like reaction due to acute hepatitis B infection in a previously fully-immunized adult. This diagnosis should be kept in mind even in the clinical scenario of a fully-immunized patient.

## Introduction

Serum sickness-like syndrome is a known extrahepatic manifestation of hepatitis B infection. This presents with a fever, erythematous skin rash, myalgias, arthralgias, fatigue and malaise. It has been estimated to develop in 10-20% of patients with acute hepatitis B infection [1]. The pathogenesis of the syndrome is hypothesized to be due to circulating immune complexes composed of hepatitis B surface antigen (HBsAg) with subsequent consumption of complement [2]. The presence of normal complement levels, however, does not rule out a diagnosis of serum sickness like syndrome secondary to acute hepatitis B infection [3].

## Case presentation

**Figure 1:**
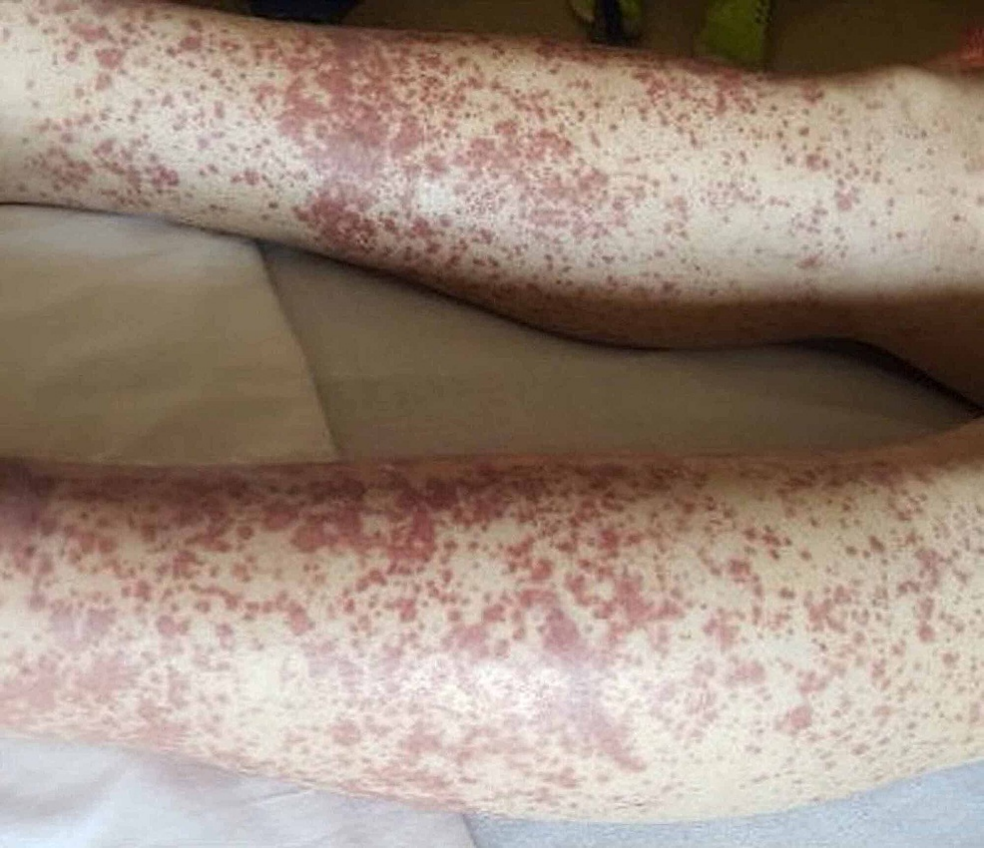
Non-blanching, erythematous macular rash on lower extremities

We describe a 36-year-old male with a two-week history of swelling, stiffness and tenderness of the knees, ankles, bilateral metacarpophalangeal (MCP) and proximal interphalangeal (PIP) joints. He also presented with three days of a painful, pruritic rash on his feet, with cephalic spread to involve lower abdomen and upper limbs. The patient attempted self management with daily naproxen and two days of oral trimethoprim-sulfamethoxazole with no improvement. The patient has medical history of essential hypertension, active intravenous heroin drug use and participated in sharing needles. He also has chronic untreated hepatitis C acquired two years prior while incarcerated. The patient had completed vaccination series for hepatitis A and hepatitis B, more than a year prior to the development of his presenting symptoms. He identified as heterosexual and denied sexual activity in the last six months or history of travel in the preceding months. He was living with a male friend who had chronic hepatitis B infection. On examination, the patient was afebrile 36.7 C, blood pressure 142/87 mmHg, respiratory rate of 18 breaths a minute, heart rate 104 beats a minute. He was able to ambulate with discomfort but without support. Minimal effusion and tenderness was noted in both knees, with bilateral ankle tenderness and restricted motion; point tenderness over the cervical spine and bilateral symmetric MCP and PIP tenderness was also noted. No joint deformity was present. Tender, non-blanching erythematous macular rash was noted over the bilateral lower extremities, extending to the penis, scrotum, lower abdomen and upper extremities, with sparing of the palms and soles (Figure 1). Initial examination was negative for jaundice, abdominal distension and ascites. Preliminary workup was remarkable for elevated liver enzymes [aspartate transaminase (AST) 375, alanine transaminase (ALT) 502], mildly elevated erythrocyte sedimentation rate (ESR) (40), C-reactive protein (CRP) (13), normal international normalised ratio (INR) (1.09), normal creatinine (Cr) [blood urea nitrogen (BUN) 16, Cr 0.5)]. He was initially managed supportively with methadone for pain, with a working diagnosis of undifferentiated vasculitis. Two days into his admission, the patient developed abdominal pain, nausea, vomiting and anorexia. At this point, examination was positive for diffuse abdominal tenderness without hepatosplenomegaly. Differential diagnosis included cryoglobulinemia secondary to chronic hepatitis C, contaminated heroin with an unknown agent causing vasculitis, acute hepatitis B infection, HIV infection and autoimmune vasculitis. Further investigative workup was ordered including complement levels, rheumatoid factor, cryoglobulins, hepatitis A IgM, hepatitis B serologies, 4th generation Ag/Ab HIV, quantitative and qualitative hepatitis C RNA, hepatitis C antibody, urinalysis (UA) and blood cultures. Results showed negative cryoglobulins, antineutrophil cytoplasmic antibody (ANCA), UA, blood culture and hepatitis A IgM; low C4 and normal C3 complements, quantitative hepatitis C virus (HCV) RNA PCR 32 IU/ml. Hepatitis B panel was positive for core antibody IgM, surface antigen, and e antigen. Hepatitis B virus (HBV) RNA PCR viral load was 214 million IU/ml. A diagnosis of serum sickness-like reaction associated with acute hepatitis B infection was consistent with clinical features, risk factors and laboratory parameters. The patient left the hospital against medical advice, having received symptomatic treatment with methadone prior to leaving. At follow-up eight months later, the patient’s symptoms had resolved. He remained positive for hepatitis B core antibody, hepatitis B e antibody and tested negative for hepatitis B surface antigen and hepatitis B surface antibody. The follow-up hepatitis B panel indicates either a resolved infection of hepatitis B, or a low-level chronic infection with hepatitis B. Follow-up laboratory assessment two years later revealed the same serology results, with the exception of hepatitis B core antibody which was not obtained at the time. The patient's HBV DNA quantitative level was <10 IU/ml during this follow-up.

## Discussion

Serum sickness-like reaction (SSLR) has been described in about 10%-20% of patients with acute hepatitis B infection [1]. The disease clinically manifests as a prodromal illness with symptoms ranging from erythematous macular or maculopapular rash to arthralgia with or without joint swelling. Hepatitis B surface antigen (HBsAg), hepatitis B core antigen (HBcAg) and viral DNA combine with antibodies to form immune complexes. These circulating immune complexes interact with complement proteins and contribute to the inflammatory phenomenon [2]. Typically, low levels of complement proteins are expected with serum sickness, however normal levels of C3 and C4 might be observed on admission [3]. Failure to attain seroconversion after complete hepatitis B vaccination is the most likely reason for our patient’s positive hepatitis B serology and clinical presentation.

Our patient presented with typical symptoms, however, suspicion was initially low for acute hepatitis B infection, given that he had documented hepatitis B vaccination. The patient also had evidence of chronic hepatitis C infection, however, his viral load was low and we considered it to be unlikely that he would develop SSLR from his chronic HCV infection, since he was not immune compromised. Aiding us in this diagnosis was our further probing into the patient's history of intravenous drug use and sharing of needles with a roommate who had chronic hepatitis B infection. The patient's symptoms of rash and arthralgias subsided with resolution of acute hepatitis B infection, further making the case for a hepatitis B-related immune phenomenon. It is possible to acquire hepatitis B infection despite complete vaccination series. Five percent of immunocompetent people are estimated to be non-responders, a figure estimated to be greater among the immunocompromised who tend to have blunted responses [4]. Serum sickness-like reaction as the initial presentation of acute hepatitis B infection in a person who has completed vaccination is uncommon but should remain in the clinician’s differential.

## Conclusions

It is important to keep in mind atypical ways in which hepatitis B may present, including extrahepatic manifestations in a patient who has been fully vaccinated.
